# Paracetamol hypersensitivity: a 5 year review

**DOI:** 10.1186/2045-7022-4-S3-P70

**Published:** 2014-07-18

**Authors:** Daniela Malheiro, Maria Sousa, Ana Moreira, Susana Cadinha, JP Moreira Silva

**Affiliations:** 1 Centro Hospitalar Vila Nova de Gaia/Espinho, Allergy Department, Portugal

## Background

Paracetamol is a widely used analgesic and antipyretic drug. HS (hypersensitivity reactions) to paracetamol are rare but appear to be increasing. Our aim was to characterize a series of patients with suspected PHS (paracetamol HS).

## Methods

A total of 245 patients with suspected NSAIDs HS were referred to our Drug Allergy Clinic from 2009 to 2013 and those with suspected PHS were evaluated. Demographic data, atopy, comorbidities, HS to other NSAIDs, timing of reaction, clinical manifestations and diagnostic procedures were assessed. PHS was confirmed by a positive DPT (drug provocation test) and considered probable based only on a suggestive clinical history.

## Results

Fifty patients (20.4%) had suspected PHS: 56% male, median age 41.5 years [6-81]; 46% atopic; 18% had chronic urticaria and/or angioedema, 18% had asthma and rhinitis and 16% had rhinitis/rhinoconjunctivitis. Thirty seven patients (74%) had suspected HS to multiple NSAIDs and 38% to other drugs, mostly antibiotics. Cutaneous reactions were reported by 60% of the subjects, both cutaneous and respiratory symptoms by 24%, anaphylaxis by 10% and isolated respiratory symptoms by 6%. Thirty one (62%) reported IR (immediate reactions), 32% DR (delayed reactions) and 6% were unable to recall the onset of the reaction. SPT and IDT were negative in all patients tested (17 out of 31 with IR; 1 out of 3 with indeterminate reaction); patch tests were negative in 9 out of 16 patients with DR. DPT with Paracetamol was performed in 60% of the patients and was positive in 4 (3 out of 18 with IR; 1 out of 10 with DR). Long term challenge was negative in 10 patients (9 with DR and negative DPT; 1 with indeterminate reaction and negative DPT). PHS was confirmed in 4 (8%) patients, excluded in 32 (64%) and considered probable in 4 (8%). The study was inconclusive in 10 (20%) because patients refused DPT or missed the scheduled appointments. It was possible to confirm HS to other NSAIDs in 1 patient with confirmed PH and in 3 patients in whom PHS was excluded. HS to other NSAIDs was considered probable in 2 patients with confirmed PHS and in 12 patients with excluded PHS.

## Conclusion

As previously described in the literature, our study suggests that PHS is rare and usually presents with cutaneous symptoms. We observed a predominance of male gender, unlike published reports on NSAIDs HS. Although negative, skin tests did not exclude PHS and DPT remains the gold standard for PHS diagnosis.

